# Analysis of SIRT1 Expression in Plasma and in an *In Vitro* Model of Preeclampsia

**DOI:** 10.1155/2020/4561083

**Published:** 2020-04-28

**Authors:** Sarah Viana-Mattioli, Priscila Nunes, Ricardo Cavalli, Valeria Sandrim

**Affiliations:** ^1^Department of Pharmacology, Institute of Biosciences of Botucatu, Universidade Estadual Paulista (UNESP), Distrito Rubiao Junior, Botucatu, São Paulo 18680-000, Brazil; ^2^Medical School, Botucatu, Universidade Estadual Paulista (UNESP), Distrito Rubiao Junior Botucatu, São Paulo 18680-000, Brazil; ^3^Department of Gynecology and Obstetrics, Faculty of Medicine of Ribeirao Preto, University of Sao Paulo, Ribeirao Preto, São Paulo 14049-900, Brazil

## Abstract

Preeclampsia (PE) is a pregnancy-specific disorder that affects 3–8% expecting mothers worldwide being one of the main causes of maternal and fetal morbidity and mortality. The search for altered circulating molecules in PE is an important target to better understand the pathophysiology of this disease. Therefore, we evaluated Sirtuin-1 (SIRT1) concentration in plasma from healthy pregnant (HP) women, gestational hypertensive women (GH), and preeclampsia women (PE) via enzyme-linked immunosorbent assay (ELISA). We also measured intracellular SIRT1 in HUVECs incubated with plasma from PE patients compared to HP and GH via Western Blot Assay. Statistical differences were considered when *p* < 0.05. SIRT1 was downregulated in PE compared to HP and GH, both in plasma and in *in vitro* assay. Similarly, SIRT1 was also reduced in pregnant women who subsequently developed PE (case) compared to women who had healthy pregnancies (control). This reduction may be indicative of possible underlying pathophysiology mechanisms in PE.

## 1. Introduction

Preeclampsia (PE) is a multifactorial untreatable maternal syndrome characterized by the onset of hypertension after 20 weeks of gestation associated with proteinuria or other end-organ damage [1]. It is indicated as a major cause of maternal and perinatal morbidity and mortality worldwide affecting an estimated 3–8% of all pregnancies [2]. Risk factors include family history, maternal age, chronic hypertension, smoking, number of pregnancies, *in vitro* fertilization, diabetes, chronic kidney disease, obesity, and multifetal gestations [3]. Although there have been many efforts to better comprehend the exact pathological mechanisms of this condition, the complexity of this disorder hampers the discovery of specific biological processes related to the disease, and, so far, the exact cause and cure of this disease have not been discovered. Therefore, placenta delivery is the only existing effective treatment [4].

The oxidative stress imbalance, an asymmetry between the intracellular reactive oxygen species (ROS) levels and the antioxidant system, is indicated as one of the main participants of the pathophysiology of PE and is known to lead to endothelial dysfunction associated with decreased nitric oxide (NO) bioavailability leading to endothelium dysfunction, a feature of the disease [5–7]. Sirtuin 1 (SIRT1), a NAD^+^-dependent class III of histone deacetylase protein, has a fundamental role in regulating oxidative stress in the vascular endothelium through its vast network of deacetylase interactions, in addition to increasing the bioavailability of NO through the activation of the endothelial nitric oxide synthase (eNOS) [8]. It is also known that this protein is downregulated by oxidative stress [9, 10].

Only three studies have evaluated SIRT1 levels in PE. One has shown that SIRT1 is reduced in trophoblasts during the last trimester of established PE [11], another study showed that SIRT1 mRNA expression is reduced in PE placentas vs. normal pregnancy and intrauterine growth restriction (IUGR) placentas [12], and the last study showed that this protein was also reduced in Human Umbilical Endothelial Cells (HUVECs) incubated with preeclamptic serum when compared to HUVEC basal control [13]. This assay is an established *in vitro* model of PE and allows us to study the effect of circulating factors released from the placenta on endothelial cells [14, 15]. Although circulating SIRT1 has been reported in other diseases [16–19], this data is missing in PE.

Then, we aimed (1) to quantify these levels in pregnant women already diagnosed with PE and compared to HP, (2) to verify a potential use of this biomarker as a predictor of PE development, and (3) to study the effect of plasma collected from PE on intracellular SIRT1 produced by endothelial cells (*in vitro* model).

## 2. Materials and Methods

### 2.1. Patients

We collected 77 plasma samples from healthy pregnant women (HP), 70 plasma samples from pregnant women with gestational hypertension (GH), and 76 plasma samples of preeclamptic (PE) collected at the Hospital das Clinicas of Ribeirao Preto. This study was approved by the Research Ethics Committee of the Faculty of Medicine of Ribeirao Preto, Brazil (reference 4682/2006, June 20th, 2006), following the principles of the Declaration of Helsinki. Exclusion criteria were pregnancy of twins, chronic hypertension, hemostatic abnormalities, diabetes mellitus, fetal abnormalities, cancer, and cardiovascular, autoimmune, renal, and hepatic diseases.

For the *in vitro* assay, we selected 10 women from each of the previous groups for the pool preparation. Exclusion criteria were >30 years old, smokers, and black women. For this part of the study, we used blood collected in standard Vacutainer tubes (Becton-Dickinson), containing heparin. The data from the in vitro assay women is shown in the supplementary file (Table S1). To verify the levels of plasma SIRT1 in pregnant before the clinical onset of PE, we measured this biomarker in a subset of samples of the court BRISA (Brazilian Ribeirao Preto and São Luis Birth Cohort Studies) [20, 21], approved by the Institutional Review Board of the HCFMRP-USP (reference 4116/2008, approved date November 11, 2008, following the principles of the Declaration of Helsinki) in which pregnant women in their first trimester (until 20 weeks of gestation) were recruited from hospitals and healthcare units to University Hospital of the Ribeirao Preto Medical School, University of São Paulo (HCFMRP-USP), Ribeirao Preto, Brazil. Clinical evaluation and blood collection were performed during the second trimester of gestation (at 20–25 weeks). Of all 1,400 pregnant women, 460 gave birth in other hospitals and from the remaining 940 pregnant women, 30 developed PE and 90 healthy participants were randomly selected as controls. For this study, we were able to include only 17 samples from women who developed PE and 17 samples of healthy pregnant women, because the other plasma samples were no longer available to properly perform the experiment.

In all studies, diagnostic criteria for PE were defined by the American College of Obstetricians and Gynecologists (ACOG) [1]. All volunteers received detailed clarification about the project and signed the informed consent form. Of these, venous blood was collected through appropriate procedures in standard Vacutainer tubes (Becton-Dickinson), containing EDTA or heparin (*in vitro* study). Then, the tubes were centrifuged at room temperature for 10 minutes at 3,200g and 1,000 *μ*L, and plasma aliquots were separated and stored together at -80°C until use.

### 2.2. *In Vitro* Assay

Human umbilical vein endothelial cells (HUVECs) (EA.hy926) were cultured at 37°C in 5% CO_2_ in DMEM medium (Gibco, CA, USA) supplemented with 10% (*v*/*v*) fetal bovine serum (SFB) (Gibco, CA, USA), 50 *μ*g/mL penicillin, 100 *μ*g/mL streptomycin, and 0.5 *μ*g/mL amphotericin B (Gibco, CA, USA). Next, we replanted 5 × 10^5^ HUVECs in 6 well plates (Jet Biofil) and after reaching 90–100% confluence, HUVECs were incubated in the medium (without phenol red and FBS) containing 10% (*v*/*v*) plasma pool of HP, GH, and PE (*N* = 10/group) for 24 hours.

Independent duplicates of each group (same cell line, same procedures, and the same pools of plasma in different experiments) were used to rule out any differences that could occur due to some particular characteristics of each cultivation, helping to consider the possible variables in cell culture procedure or protein extraction procedure. The protein concentration used was 20 *μ*g for all samples.

### 2.3. SIRT1 Measurement

The intracellular measurement of STIR1 was performed using cell (HUVECs) lysates using RIPA lysis buffer (Sigma-Aldrich, Saint Louis, MO, USA) with protease inhibitor cocktail (Sigma-Aldrich, Saint Louis, MO, USA). We used the Bradford method to measure protein concentration. Then, equal amounts of protein were separated on 4-12% Bolt Bis-Tris Plus acrylamide gel (Invitrogen, Thermo Fisher Scientific, California, USA) and blotted onto nitrocellulose membranes. The membranes were blocked for 1.5 hours with 5% nonfat dry milk diluted in TBS (1 M Tris, 5 M NaCl, pH 7.2, 500 *μ*L Tween-20) and incubated with primary antibodies against SIRT1 (1 : 400, Santa Cruz Biotechnology, Inc.), and *β*-actin (1 : 1000, Santa Cruz Biotechnology, Inc.) for 2 h, as well as the secondary Donkey Anti-Rabbit IgG (HRP) antibody (Abcam, Cambridge, UK) (1 : 75000). The immunoreactive components were developed using the Luminol SuperSignal West Femto Maximum Sensitivity Substrate reagent (Pierce Biotechnology, Massachusetts, USA) in a G-BOX photodocumenting system (Syngene International Limited). The assay to control nonspecific markings was performed with Donkey Anti-Rabbit IgG (HRP) secondary antibody incubation. We analyzed the results at ImageJ (National Institutes of Health).

Plasma SIRT1 concentration was evaluated using the commercial ELISA kit (Human SIRT1 ELISA Kit, RayBiotech, Peachtree Corners, GA, USA). Optical density was determined at 450 nm using a microplate reader (Synergy 4, BioTek, VT, USA).

### 2.4. Statistical Analysis

Statistical analysis was performed with GraphPad Prism for Windows, version 5.0 (GraphPad Software, San Diego, CA, USA). Categorical variables were compared by the Student *t*-test or ANOVA followed by the Tukey test (for normally distributed variables) or Mann-Whitney's test and Kruskal-Wallis followed by Dunn's multiple comparison test (for not normally distributed variables). For all tests, a probability value of *p* < 0.05 (two-tailed) was considered significant.

**Figure 1:**
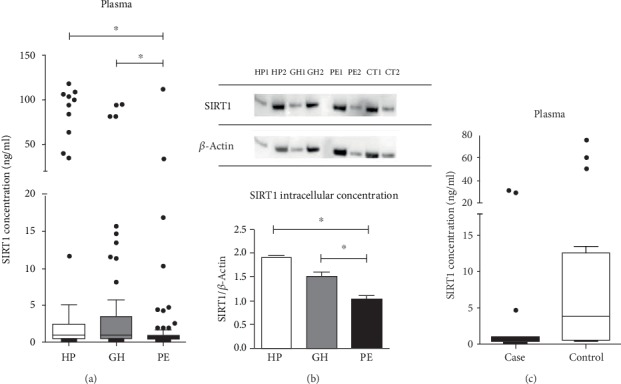
(a) Plasma SIRT1 concentration from healthy (HP, *n* = 77), gestational hypertensive (GH, *n* = 70), and preeclamptic pregnant (PE, *n* = 76) women. SIRT1 concentration found in plasma was reduced in PE vs. GH and HP controls (*p* < 0.001). (b) Intracellular SIRT1 concentration from HUVECs incubated with pools of healthy (HP, *n* = 10), gestational hypertensive (GH, *n* = 10), and preeclamptic pregnant (PE, *n* = 10) women with plasma 10% (*v*/*v*). SIRT1 concentration was reduced in HUVECs incubated with PE vs. HP and GH (*p* < 0.05 and *p* < 0.001, respectively). HUVECs with no plasma treatment were used as control (CT). Independent duplicates of each group were used to rule out any differences that could occur due to some characteristic of each cultivation, helping to consider the possible variables in cell culture procedure (1 is for the first experiment and 2 for the replicated experiment). The protein concentration used was 20 *μ*g for all samples, and final values are mean, S.D. from samples 1 and 2 from each group. (c) SIRT1 plasmatic concentration from pregnant women who subsequently developed PE (case) compared to women who had healthy pregnancies (control). SIRT1 concentration was reduced in case vs. control women (*p* < 0.001). A comparison of concentration among groups was by the Kruskal-Wallis test followed by the Dunn multiple comparison test for the comparison between HP, GH, and PE. For the *in vitro* assay, we used one-way ANOVA followed by Tukey's multiple comparison test. Nonparametric *t*-tests were performed for comparisons between case and control groups (*p* < 0.05). For illustrative purposes, we chose to represent the analysis in the Tukey method for Box Whiskers; dots are 1.5 times the interquartile distance or to the highest or lowest point, whichever is shorter.

For illustrative purposes, we chose to represent the ELISA analysis in the Tukey method for Box Whiskers; dots are 1.5 times the interquartile distance or to the highest or lowest point, whichever is shorter (Figures 1(a) and 1(c)).

## 3. Results

General clinical parameters of healthy pregnant women, gestational hypertensive pregnant women, and preeclamptic women whose plasma samples were collected and used to perform the plasma SIRT1 concentration analysis are shown in Table 1. No differences were observed in maternal age and gestational age at sampling. Body mass index (BMI), systolic blood pressure (SBP), and diastolic blood pressure (DBP) were increased in PE and GH groups compared with the HP group. Gestational age (GA) at delivery was lower in PE compared with HP and GH. Newborn weight was lower in GH and PE when compared to HP and reduced in PE when compared to GH.

While investigating SIRT1 plasmatic concentration (Figure 1(a)), we found this protein was reduced in PE when compared to HP and GH (*p* < 0.001). Additionally, the clinical features from women from Table 1 showed an increased BMI in PE when compared to the HP group, which is considered a risk factor in the development of PE [3]. Thereupon, we have further investigated this result to verify if there was a correlation between BMI and SIRT1 plasmatic levels (supplementary file Table S2). No correlations were found (*p* < 0.05).

Interestingly, in the *in vitro* model, intracellular SIRT1 was reduced in the HUVECs incubated with PE plasma in comparison with HP and GH (*p* < 0.05 and *p* < 0.001, respectively) (Figure 1(b)).

Based on these results, we aimed to further investigate the SIRT1 levels in the development of PE by analyzing the plasma of pregnant women *collected before* developed PE (case) compared to women who had healthy pregnancies (control). The clinical data from the case vs. control study are shown in Supplementary file (Table S3). Importantly, we found that SIRT1 levels were reduced in pregnant who subsequently develop preeclampsia (case) compared to control (*p* < 0.05) (Figure 1(c)).

## 4. Discussion

To our knowledge, we are the first group to demonstrate that SIRT1 plasmatic concentration is lowered in preeclamptic women plasma in comparison to healthy and gestational hypertensive pregnant in a sizeable cohort study, with at least 70 women per group. Moreover, we demonstrated that the incubation of HUVECs, from a healthy cell line, with plasma from these groups was able to reproduce the in vivo results in intracellular protein expression levels. Finally, this is the first study that suggests that SIRT1 reduction, at 20-25 weeks of gestation, may indicate PE development, emerging SIRT1 as a possible biomarker of this pathology.

Corroborating with our results, one study investigated SIRT1 in the placenta showing its reduction in trophoblasts during the last trimester of established PE compared to control (*p* = 0.004) [11], another study showed that SIRT1 mRNA expression is reduced in PE placentas vs. normal pregnancy and intrauterine growth restriction (IUGR) placentas (*p* < 0.001) [12], and the last study showed that this protein was also reduced in Human Umbilical Endothelial Cells (HUVECs) incubated with PE serum (*n* = 2) when compared to HUVEC basal control (*p* < 0.05 vs. control), but no healthy pregnant controls were used [13].

Although oxidative stress is considered a common feature of pregnancy [22], it is greatly increased in PE by reduced antioxidant capacity in these women organisms [23]. This condition has also been related to the reduction of SIRT1 levels and activity [24]. For example, previous studies have demonstrated that oxidative stress can induce the activation of JNK1, which phosphorylates SIRT1 on its Ser27, Ser47, and Thr530 residues, increasing SIRT1 activation [25, 26]. However, during oxidative imbalance condition, the constant activation of SIRT1 by JNK1 leads to SIRT1 degradation via ubiquitination [27]. ROS also induces the expression of miR-34a, and this miR binds to SIRT1 mRNA on the 3′UTR region inhibiting this protein expression [28]. Furthermore, oxidative stress can oxidase SIRT1 cysteine residues reducing its activity and promoting its degradation in the proteasomes [29], besides depleting NAD+ and consequently reducing SIRT1 activity [30]. Given the SIRT1 role in regulating cell metabolism, angiogenesis, inflammation, apoptosis, DNA repair, and stress resistance [31], it is possible that this reduction may play a pivotal role in PE. Previous studies showed that the activation of SIRT1 by resveratrol was able to reduce the soluble fms-like tyrosine kinase 1 (sFlit-1) secretion in preeclamptic placentas [32] and trophoblasts [33, 34] reducing this molecule antiangiogenic effects in PE pathogenesis. Another study showed that the downregulation of SIRT1 levels in gestational tissues (after labor placenta, amnion, and choriodecidua) might be responsible for the augmented NF-*κ*B transcriptional activity and reduction of PPAR, the augmentation of proinflammatory cytokines and matrix metalloproteinase 9 (MMP-9) expression, which are all fundamental characteristics in human labor [20].

The oxidative stress in PE is also known to lead to endothelial impairment, and as shown in our *in vitro* results, this disability might be related to the reduction in SIRT1 intracellular expression. Indeed, it has been reported that SIRT1 plays a pivotal role in combating the vascular endothelium oxidative stress through a complex signaling network that involves the deacetylation/regulation of FOXOs, NFKB, NOX, SOD, and eNOS [8].

Additionally, since previous large cohort studies have shown that SIRT1 genetic variations are related to increases in BMI and risk of obesity [21, 35], our investigation on the correlation between BMI and SIRT1 plasmatic levels (Table S3) showed no correlation between these two parameters, proposing that they might not be dependently related to PE pathophysiology. Supporting our hypothesis that SIRT1 reduced the level itself is one of the main participants in the development of PE, our results also showed that there was no difference between GH and PE BMI levels, but SIRT1 plasmatic levels were decreased in PE compared to this group. Our *in vitro* and case vs. control study results reinforce showing the same results even though BMI is not significantly increased in GH and PE compared to HP and between case and control groups. However, this does not exclude the possibility that SIRT1 genetic variation might be related to the increased BMI in these women and further studies are necessary to evaluate this aspect.

In these contexts, to reduce the oxidative stress may be an important target in established PE therapy. Indeed, some studies that focused on the use of antioxidants, such as vitamins C and E, were not successful [36, 37]. However, resveratrol, a potent natural antioxidant and SIRT1 activator presented mainly in grapes and wine, emerges as a possible supporter to attenuate PE symptoms as it has been shown by our group [38] and others [33, 39–42]. Nevertheless, more research on the side effects of the use of SIRT1 activators in humans is necessary given previous reports of its tissue-dependent activity [43, 44].

Finally, our case-control *in vivo* study indicates that this protein may be a biomarker for the increased risk of PE at the beginning of the disease. This finding requires validation of larger cohort studies and evaluation through randomized controlled trials [45]. Therefore, our study cannot be considered a predictive study but may be considered guidance for the next studies, being the first one to demonstrate that SIRT1 plasmatic concentration is lowered in women who subsequently developed PE plasma in comparison to women who had normal gestations at 20–25-week interval.

## 5. Conclusions

In conclusion, SIRT1 plasmatic concentration is lowered in preeclamptic women in comparison to healthy pregnant and gestational hypertensive pregnant. Likewise, the intracellular protein expression results were the same in HUVECs incubated with these groups of plasma. SIRT1 was also reduced in the plasma of pregnant women who subsequently developed PE (case) compared to women who had normal pregnancies (control). These results may indicate an important underlying pathophysiology mechanism, and a treatment target for future studies helping the monitoring of these patients, and possibly decreasing mortality, as well as symptoms of the maternal syndrome in PE.

## Figures and Tables

**Table 1:** Clinical characteristics of healthy, gestational hypertensive, and preeclamptic pregnant women enrolled in this study.

Parameters	HP (*n* = 77)	GH (*n* = 70)	PE (*n* = 76)
GA at sampling (weeks)	36 ± 3	36 ± 5	34 ± 5
Maternal age (years)	26 ± 6	27 ± 7	28 ± 6
BMI (kg/m^2^)	27 ± 5	34 ± 5^∗^	34 ± 5^∗^
SBP at sampling (mmHg)	110 ± 1	130 ± 1^∗^	140 ± 1^∗^
DBP at sampling (mmHg)	74 ± 8	82 ± 10^∗^	82 ± 10^∗^
GA at delivery (weeks)	40 ± 2	39 ± 2	37 ± 4^∗^
Newborn weight (g)	3,255 ± 646	3,138 ± 554^∗^	2,644 ± 901^∗^
*α*-Methyldopa at sampling (%)	NA	70	88
Nifedipine at sampling (%)	NA	7	21
Hydralazine at sampling (%)	NA	4	4

Values are the means ± S.D. or percentage. HP: healthy pregnant; GH; gestational hypertension; PE: preeclampsia; GA: gestational age; BMI: body mass index; SBP: systolic blood pressure; DBP: diastolic blood pressure; NA: not applicable. ^∗^*p* < 0.05 vs. healthy pregnant.

## Data Availability

The data used to support the findings of this study are available from the corresponding author upon request.

## Supplementary Materials

Supplementary Materials Table S1. General characteristics of patients enrolled in the in vitro study. Table S2. Correlation between BMI and SIRT1 concentration in plasma samples from HP, GH, and PE women. Table S3. General characteristics of patients enrolled in the Case vs Control study.

## References

[1] American College of Obstetricians and Gynecologists; Task Force on Hypertension in Pregnancy (2013). Hypertension in pregnancy. Report of the American College of Obstetricians and Gynecologists’ Task Force on Hypertension in Pregnancy. *Obstetrics & Gynecology*.

[2] Armaly Z., Jadaon J. E., Jabbour A., Abassi Z. A. (2018). Preeclampsia: novel mechanisms and potential therapeutic approaches. *Frontiers in Physiology*.

[3] Phipps E. A., Thadhani R., Benzing T., Karumanchi S. A. (2019). Pre-eclampsia: pathogenesis, novel diagnostics and therapies. *Nature Reviews. Nephrology*.

[4] Dekker G. A. (2014). Management of preeclampsia. *Pregnancy Hypertension: An International Journal of Women's Cardiovascular Health*.

[5] Mutlu-Türkoglu U., Ademoglu E., Ibrahimoglu L., Aykaç-Toker G., Uysal M. (1998). Imbalance between lipid peroxidation and antioxidant status in preeclampsia. *Gynecologic and Obstetric Investigation*.

[6] Sankaralingam S., Xu H., Davidge S. T. (2010). Arginase contributes to endothelial cell oxidative stress in response to plasma from women with preeclampsia. *Cardiovascular Research*.

[7] Sandrim V. C., Montenegro M. F., Palei A. C. T. (2010). Increased circulating cell-free hemoglobin levels reduce nitric oxide bioavailability in preeclampsia. *Free Radical Biology & Medicine*.

[8] Zhang W., Huang Q., Zeng Z., Wu J., Zhang Y., Chen Z. (2017). Sirt1 inhibits oxidative stress in vascular endothelial cells. *Oxidative Medicine and Cellular Longevity*.

[9] Guo H., Chen Y., Liao L., Wu W. (2013). Resveratrol protects HUVECs from oxidized-LDL induced oxidative damage by autophagy upregulation via the AMPK/SIRT1 pathway. *Cardiovascular Drugs and Therapy*.

[10] Yang Y., Duan W., Lin Y. (2013). SIRT1 activation by curcumin pretreatment attenuates mitochondrial oxidative damage induced by myocardial ischemia reperfusion injury. *Free Radical Biology & Medicine*.

[11] Broady A. J., Loichinger M. H., Ahn H. J., Davy P. M. C., Allsopp R. C., Bryant-Greenwood G. D. (2017). Protective proteins and telomere length in placentas from patients with pre- eclampsia in the last trimester of gestation. *Placenta*.

[12] Poidatz D., Dos Santos E., Duval F. (2015). *Involvement of estrogen-related receptor-γ and mitochondrial content in intrauterine growth restriction and preeclampsia*. *Fertility and Sterility*.

[13] Yin Y., Feng Y., Zhao H. (2017). SIRT1 inhibits releases of HMGB1 and HSP70 from human umbilical vein endothelial cells caused by IL-6 and the serum from a preeclampsia patient and protects the cells from death. *Biomedicine & Pharmacotherapy*.

[14] Sandrim V. C., Dias M. C., Bovolato A. L. . C., Tanus-Santos J. E., Deffune E., Cavalli R. C. (2016). Plasma from pre-eclamptic patients induces the expression of the anti-angiogenic miR-195-5p in endothelial cells. *Journal of Cellular and Molecular Medicine*.

[15] Calicchio R., Buffat C., Mathieu J. R. (2013). Preeclamptic plasma induces transcription modifications involving the AP-1 transcriptional regulator JDP2 in endothelial cells. *The American Journal of Pathology*.

[16] Kumar R., Chaterjee P., Sharma P. K. (2013). Sirtuin1: a promising serum protein marker for early detection of Alzheimer’s disease. *PLoS ONE*.

[17] Kumar R., Mohan N., Upadhyay A. D. (2014). Identification of serum sirtuins as novel noninvasive protein markers for frailty. *Aging Cell*.

[18] Mariani S., Fiore D., Basciani S. (2015). Plasma levels of SIRT1 associate with non-alcoholic fatty liver disease in obese patients. *Endocrine*.

[19] Zbroch E., Bazyluk A., Malyszko J. (2020). The serum concentration of anti-aging proteins, Sirtuin1 and *α*Klotho in patients with end-stage kidney disease on maintenance hemodialysis. *Clinical Interventions in Aging*.

[20] Lappas M., Mitton A., Lim R., Barker G., Riley C., Permezel M. (2011). SIRT1 is a novel regulator of key pathways of human labor. *Biology of Reproduction*.

[21] Peeters A. V., Beckers S., Verrijken A. (2008). Association of SIRT1 gene variation with visceral obesity. *Human Genetics*.

[22] Belo L., Caslake M., Santos-Silva A. (2004). LDL size, total antioxidant status and oxidised LDL in normal human pregnancy: a longitudinal study. *Atherosclerosis*.

[23] Powe C. E., Levine R. J., Karumanchi S. A. (2011). Preeclampsia, a disease of the maternal endothelium: the role of anti-angiogenic factors and implications for later cardiovascular disease. *Circulation*.

[24] Salminen A., Kaarniranta K., Kauppinen A. (2013). Crosstalk between oxidative stress and SIRT1: impact on the aging process. *International Journal of Molecular Sciences*.

[25] Kamata H., Honda S.-I., Maeda S., Chang L., Hirata H., Karin M. (2005). *Reactive Oxygen Species Promote TNFα-Induced Death and Sustained JNK Activation by Inhibiting MAP Kinase Phosphatases*. *Cell*.

[26] Nasrin N., Kaushik V. K., Fortier E. (2009). JNK1 phosphorylates SIRT1 and promotes its enzymatic activity. *PLoS One*.

[27] Gao Z., Zhang J., Kheterpal I., Kennedy N., Davis R. J., Ye J. (2011). Sirtuin 1 (SIRT1) protein degradation in response to persistent c-Jun N-terminal kinase 1 (JNK1) activation contributes to hepatic steatosis in obesity. *The Journal of Biological Chemistry*.

[28] Yamakuchi M., Ferlito M., Lowenstein C. J. (2008). miR-34a repression of SIRT1 regulates apoptosis.

[29] Caito S., Rajendrasozhan S., Cook S. (2010). SIRT1 is a redox-sensitive deacetylase that is post-translationally modified by oxidants and carbonyl stress. *The FASEB Journal*.

[30] Furukawa A., Tada-Oikawa S., Kawanishi S., Oikawa S. (2007). H2O2 accelerates cellular senescence by accumulation of acetylated p53 via decrease in the function of SIRT1 by NAD+ depletion. *Cellular Physiology and Biochemistry*.

[31] Giblin W., Skinner M. E., Lombard D. B. (2014). Sirtuins: guardians of mammalian healthspan. *Trends in Genetics*.

[32] Cudmore M. J., Ramma W., Cai M. (2012). Resveratrol inhibits the release of soluble fms-like tyrosine kinase (sFlt-1) from human placenta. *Am. J. Obstet. Gynecol*.

[33] Hannan N. J., Brownfoot F. C., Cannon P. (2017). Resveratrol inhibits release of soluble fms-like tyrosine kinase (sFlt-1) and soluble endoglin and improves vascular dysfunction - implications as a preeclampsia treatment. *Scientific Reports*.

[34] Hastie R., Brownfoot F. C., Pritchard N. (2019). EGFR (epidermal growth factor receptor) signaling and the mitochondria regulate sFlt-1 (soluble FMS-like tyrosine kinase-1) secretion. *Hypertension*.

[35] Zillikens M. C., van Meurs J. B. J., Rivadeneira F. (2009). SIRT1 genetic variation is related to BMI and risk of obesity. *Diabetes*.

[36] Poston L., Briley A. L., Seed P. T., Kelly F. J., Shennan A. H. (2006). Vitamin C and vitamin E in pregnant women at risk for pre-eclampsia (VIP trial): randomised placebo-controlled trial. *Lancet*.

[37] Roberts J. M., Myatt L., Spong C. Y. (2010). Vitamins C and E to prevent complications of pregnancy-associated hypertension. *The New England Journal of Medicine*.

[38] Caldeira-Dias M., Montenegro M. F., Bettiol H. (2019). Resveratrol improves endothelial cell markers impaired by plasma incubation from women who subsequently develop preeclampsia. *Hypertension Research*.

[39] Zou Y., Zuo Q., Huang S. (2014). Resveratrol inhibits trophoblast apoptosis through oxidative stress in preeclampsia-model rats. *Molecules*.

[40] Wang P., Huang C., Gao J. (2020). Resveratrol induces SIRT1-dependent autophagy to prevent H2O2-induced oxidative stress and apoptosis in HTR8/SVneo cells. *Placenta*.

[41] Zou Y., Li S., Wu D. (2019). Resveratrol promotes trophoblast invasion in pre-eclampsia by inducing epithelial-mesenchymal transition. *Journal of Cellular and Molecular Medicine*.

[42] Ding J., Kang Y., Fan Y., Chen Q. (2017). Efficacy of resveratrol to supplement oral nifedipine treatment in pregnancy-induced preeclampsia. *Endocrine Connections*.

[43] Chen D., Bruno J., Easlon E. (2008). Tissue-specific regulation of SIRT1 by calorie restriction. *Genes & Development*.

[44] Li Y., Xu W., McBurney M. W., Longo V. D. (2008). SirT1 inhibition reduces IGF-I/IRS-2/Ras/ERK1/2 signaling and protects neurons. *Cell Metabolism*.

[45] McCarthy F. P., Ryan R. M., Chappell L. C. (2018). Prospective biomarkers in preterm preeclampsia: a review. *Pregnancy Hypertension*.

