# Supplementary material for: Analysis of SIRT1 Expression in Plasma and in an In Vitro Model of Preeclampsia
Source: Oxid Med Cell Longev. 2020 Apr 28;2020:4561083. doi: 10.1155/2020/4561083 (PMC7204130; doi:10.1155/2020/4561083)
Supplement: Supplementary Materials — Table S1. General characteristics of patients enrolled in the in vitro study. Table S2. Correlation between BMI and SIRT1 concentration in plasma samples from HP, GH, and PE women. Table S3. General characteristics of patients enrolled in the Case vs Control study. [file 4561083.f1.docx]

Supplementary Material

**Table S1.** General characteristics of patients enrolled in the *in vitro* study.

| **Parameters** | **HP (n=10)** | **GH (n=10)** | **PE (n=10)** |
| --- | --- | --- | --- |
| GA at sampling (weeks) | 37 ± 1 | 36 ± 5 | 36 ± 2 |
| Maternal Age (years) | 23 ± 5 | 20 ± 3 | 23 ± 3 |
| BMI (kg/m²) | 28 ± 4 | 37 ± 6 | 35 ± 3 |
| SBP at sampling (mmHg) | 105 ± 12 | 130 ± 12 | 135 ± 18 |
| GA at delivery (weeks) | 40 ± 2 | 39 ± 2 | 37 ± 4* |
| Newborn weight (g) | 3302 ± 382 | 3237 ± 394 | 2825 ± 738 |

Data as mean ± SD. BMI means body mass index; SBP, systolic blood pressure; DBP, diastolic blood pressure. * vs HP and GH. HP, Healthy Pregnant; GH, gestational hypertension; PE, preeclampsia

**Table S2.** Correlation between BMI and SIRT1 concentration in plasma samples from HP, GH, and PE women.

|  | **HP** | **GH** | **PE** |
| --- | --- | --- | --- |
| BMI (Kg/m^2^) | 0.17 (0.18) | 0.15 (0.19) | 0.04 (0.70) |

Data as correlation coefficient (*P* value); BMI, body mass index. HP, Healthy Pregnant; GH, gestational hypertension; PE, preeclampsia

**Table S3.** General characteristics of patients enrolled in the Case vs Control study.

| **Parameters** | **Case (n=17)** | **Control (n=17)** | **p** |
| --- | --- | --- | --- |
| GA at sampling (weeks) | 23 ± 1 | 23 ± 1 | 1.00 |
| Maternal Age (years) | 27 ± 5 | 26 ± 6 | 0.60 |
| BMI (kg/m²) | 30 ± 6 | 28 ± 3 | 0.23 |
| SBP at sampling (mmHg) | 116 ± 11 | 107 ± 8 | 0.01* |
| DBP at sampling (mmHg) | 75 ± 7 | 66 ± 6 | 0.00* |
| Nulliparous | 6 | 6 | 1.00 |
| Newborn weight (g) | 2956 ± 914 | 3401 ± 411 | 0.08 |

Data as mean ± SD or n (percentage of total). BMI means body mass index; SBP, systolic blood pressure; DBP, diastolic blood pressure; NBW, newborn weight. * vs case group. HP, Healthy Pregnant; GH, gestational hypertension; PE, preeclampsia
